# Predicting sporadic Alzheimer’s disease progression via inherited Alzheimer’s disease-informed machine-learning

**DOI:** 10.1002/alz.12032

**Published:** 2020-02-11

**Authors:** Nicolai Franzmeier, Nikolaos Koutsouleris, Tammie Benzinger, Alison Goate, Celeste M. Karch, Anne M. Fagan, Eric McDade, Marco Duering, Martin Dichgans, Johannes Levin, Brian A. Gordon, Yen Ying Lim, Colin L. Masters, Martin Rossor, Nick C. Fox, Antoinette O’Connor, Jasmeer Chhatwal, Stephen Salloway, Adrian Danek, Jason Hassenstab, Peter R. Schofield, John C. Morris, Randall J. Bateman, the Alzheimer’s disease neuroimaging initiative (ADNI), the Dominantly Inherited Alzheimer Network (DIAN), Michael Ewers

**Affiliations:** 1 Institute for Stroke and Dementia Research, Klinikum der Universität München, Ludwig-Maximilians-Universität LMU, Munich, Germany; 2 Department of Psychiatry and Psychotherapy, Ludwig-Maximilians-Universität LMU, Munich, Germany; 3 Department of Radiology, Washington University in St. Louis, St. Louis, Missouri, USA; 4 Knight Alzheimer’s Disease Research Center, Washington University in St. Louis, St. Louis, Missouri, USA; 5 Department of Genetics and Genomic Sciences, Icahn School of Medicine at Mount Sinai, New York, New York, USA; 6 Ronald M. Loeb Center for Alzheimer’s Disease, Department of Neuroscience, Icahn School of Medicine at Mount Sinai, New York, New York, USA; 7 Hope Center for Neurological Disorders, Washington University in St. Louis, St. Louis, Missouri, USA; 8 Department of Psychiatry, Washington University in St. Louis, St. Louis, Missouri, USA; 9 Department of Neurology, Washington University in St. Louis, St. Louis, Missouri, USA; 10 Munich Cluster for Systems Neurology, Munich, Germany; 11 German Center for Neurodegenerative Diseases (DZNE), Munich, Germany; 12 Department of Neurology, Ludwig-Maximilians Universität München, Munich, Germany; 13 Mallinckrodt Institute of Radiology, Washington University, St. Louis, Missouri, USA; 14 Department of Psychological and Brain Sciences, Washington University, St. Louis, Missouri, USA; 15 The Florey Institute, The University of Melbourne, Parkville, Victoria, Australia; 16 Dementia Research Centre, University College London, Queen Square, London, UK; 17 Massachusetts General Hospital, Department of Neurology, Harvard Medical School, Boston, Massachusetts, USA; 18 Department of Neurology, Warren Alpert Medical School of Brown University, Providence, Rhode Island, USA; 19 Neuroscience Research Australia, Randwick, New South Wales, Australia; 20 School of Medical Sciences, University of New South Wales, Sydney, New South Wales, Australia; 21 ADNI Consortium members are listed in the appendix; 22 DIAN Consortium members are listed in the appendix

**Keywords:** Alzheimer’s disease, autosomal-dominant Alzheimer’s disease, biomarkers, machine learning, progression prediction, MRI, PET, risk enrichment

## Abstract

**Introduction:**

Developing cross-validated multi-biomarker models for the prediction of the rate of cognitive decline in Alzheimer’s disease (AD) is a critical yet unmet clinical challenge.

**Methods:**

We applied support vector regression to AD biomarkers derived from cerebrospinal fluid, structural magnetic resonance imaging (MRI), amyloid-PET and fluorodeoxyglucose positron-emission tomography (FDG-PET) to predict rates of cognitive decline. Prediction models were trained in autosomal-dominant Alzheimer’s disease (ADAD, n = 121) and subsequently cross-validated in sporadic prodromal AD (n = 216). The sample size needed to detect treatment effects when using model-based risk enrichment was estimated.

**Results:**

A model combining all biomarker modalities and established in ADAD predicted the 4-year rate of decline in global cognition (R^2^ = 24%) and memory (R^2^ =25%) in sporadic AD. Model-based risk-enrichment reduced the sample size required for detecting simulated intervention effects by 50%–75%.

**Discussion:**

Our independently validated machine-learning model predicted cognitive decline in sporadic prodromal AD and may substantially reduce sample size needed in clinical trials in AD.

## 1 BACKGROUND

Alzheimer’s disease (AD) is characterized by amyloid-beta (A*β*) deposition, tau pathology, neurodegeneration, and cognitive decline.^1^ Diagnosing and staging AD has been greatly facilitated by in vivo biomarkers including positron-emission tomography (PET) and cerebrospinal fluid (CSF) markers of Aα and pathologic tau, as well as volumetric magnetic resonance imaging (MRI) and fluorodeoxyglucose (FDG)-PET measures of neurodegeneration.^2^ Apart from diagnosing AD, a clinically important challenge is predicting the worsening of cognitive impairment.^3^

Previous studies have shown that markers of primary AD pathology (ie, CSF A*β*_1–42_, p-tau_181_, A*β*_1–42_/p-tau_181_ ratio, amyloid-PET), neurodegeneration (structural MRI, FDG-PET), or biomarker combinations can predict future conversion from mild cognitive impairment (MCI) to AD dementia.^4–9^ The conversion from MCI to AD dementia is, however, a binary diagnostic categorization that does not capture the *rate* of cognitive change within the AD continuum.^10^ Biomarker-based point prediction of cognitive decline is, thus, a challenge to sufficiently power clinical trials via risk enrichment and to identify subjects at imminent risk of cognitive deterioration. Previous studies have shown that higher amyloid-PET,^11,12^ MRI-assessed hippocampal atrophy,^13^ FDG-PET hypometabolism,^12^ and tau levels^14,15^ are associated with faster cognitive decline. Because biomarker combinations can enhance the accuracy for predicting clinical progression,^16^ a critical yet unresolved question is how to merge multimodal and increasingly complex (eg, voxel-wise PET and MRI) data to maximize the prediction accuracy while keeping the number of assessments and costs low. Machine learning, which is instrumental for data mining,^17^ is well suited to identify biomarker sets for predicting cognitive decline. Here, recent machine-learning approaches to AD biomarker data showed that sets of imaging and CSF-derived markers discriminated MCI and AD patients from healthy controls^18^ and predicted the rate of future cognitive decline^19,20^ and time to symptom onset.^21^

A limitation of machine learning is, however, that algorithms may perform well in the sample they were trained on but rarely generalize to new data.^22,23^ To address this, previous studies have applied within-sample cross-validation (CV), in which a given sample is iteratively divided into training and test data to ensure that model training and testing are conducted on different datasets.^18,24^ While reducing the likelihood of overfitting, this approach leaves unaddressed the question whether the algorithm indeed generalizes to new and unseen data from independently recruited participants,^25^ which is considered the gold standard of evaluating machine-learning performance.

Here, we aimed to establish a biomarker-based model to predict AD-related cognitive change rates, using validation across two independently recruited, deeply phenotyped AD samples. To this end, we first trained and cross-validated a support vector regression (SVR)model using amyloid-PET, FDG-PET, structural MRI, and CSF data from 121 autosomal-dominant Alzheimer’s disease (ADAD) subjects for predicting the estimated years to symptom onset (EYO), a proxy of cognitive decline. ADAD constitutes a unique training sample as the disease onset is at a relatively young age and the development of AD pathology and cognitive decline are not confounded by age-related comorbidities (eg, TDP-43, small-vessel disease).^26^ Because in ADAD the time course of the development of dementia symptoms is strongly genetically driven, EYO can be used as a surrogate marker of AD-related cognitive decline.^27–29^ Here, we first trained biomarker-based machine learning models for predicting EYO in ADAD. After model training and CV in ADAD, we tested whether the best model predicts up to 4-year cognitive decline when applied to an independent sample of 216 individuals with sporadic prodromal AD. Last, we tested whether machine learning–based selection of “at-risk” subjects can enhance the sensitivity to detect potential intervention effects and can reduce the required sample size in intervention studies.

## 2 METHODS

### 2.1 Participants

#### 2.1.1 Dominantly Inherited Alzheimer Network (DIAN)

We included 121 carriers of AD causative *PSEN1* and *2* or *APP* mutations (MC, data freeze 10), as well as 54 non-carrier siblings as healthy reference subjects for biomarker scaling (see supporting information). Subject inclusion required availability of baseline 3T T1-structural MRI, amyloid-PET (PiB), FDG-PET, and CSF data. No selection bias (ie, for age, sex, education) was found between the included and non-included subjects. EYO were defined as the difference between a participant’s age at examination and the parental age of symptom onset. All participants provided written informed consent; local ethical approval was obtained at each participating DIAN site.

#### 2.1.2 Alzheimer’s Disease Neuroimaging Initiative (ADNI)

Three hundred ninety-one individuals meeting Petersen criteria for amnestic MCI were included from ADNI, based on availability of baseline 3T T1-structural MRI, amyloid-PET (AV45), FDG-PET, CSF, and cognitive data (ie, ADNI-MEM and ADAS13), plus at least one (and up to four) annually consecutive cognitive follow-up assessments. No selection bias for age, sex, or education was found between the included and non-included ADNI subjects. A*β*-status was determined using a pre-established global AV45-PET standard uptake value ratio (SUVR) cut-off of 1.11.^30^ Cognitively normal A*β*+ subjects were not included, due to the few subjects meeting our inclusion criteria (N = 50/46/8/0 with 1/2/3/4-year follow-up). As a healthy reference group for biomarker scaling, we included baseline data of 49 cognitively normal A*β*-negative (ie, global AV45-PET SUVR<1.11) subjects below the age of 70 scoring >28 on the Mini-Mental State Exam (MMSE).

### 2.2 Neuroimaging and biomarker assessment

Neuroimaging in DIAN and ADNI was performed using similar protocols initially developed for ADNI. Structural MRI has been recorded on 3T scanners with a spatial resolution of 1.1 × 1.1 × 1.2 mm. FDG-PET has been acquired consistently in both samples (ie, 30 to 60 minutes after tracer injection, SUVR normalization to the brainstem), whereas Amyloid-PET scanning has been conducted using PiB in DIAN (40–70 minutes after tracer injection, SUVR normalized to the whole cerebellum) and AV45 in ADNI (50–70 minutes after tracer injection, SUVR normalized to the whole cerebellum). All ADNI^31,32^ and DIAN^29^ imaging protocols have been described previously. For the current study, we used FreeSurfer processed (Version 5.1) region of interest (ROI) data (ie, cortical thickness and subcortical volumes for structural MRI and SUVR values for PET) provided by the DIAN and ADNI neuroimaging cores. CSF concentrations of A*β*_1–42_, phosphorylated tau at threonine 181 (p-tau_181_), and total tau were measured by the using multiplex xMap Luminex in DIAN and via the Elecsys cobas e 601 instrument^33^ in ADNI. Details on biomarker assessments can be found in the supporting information.

### 2.3 DIAN—support vector regression training and nested cross-validation

**FIGURE 1:**
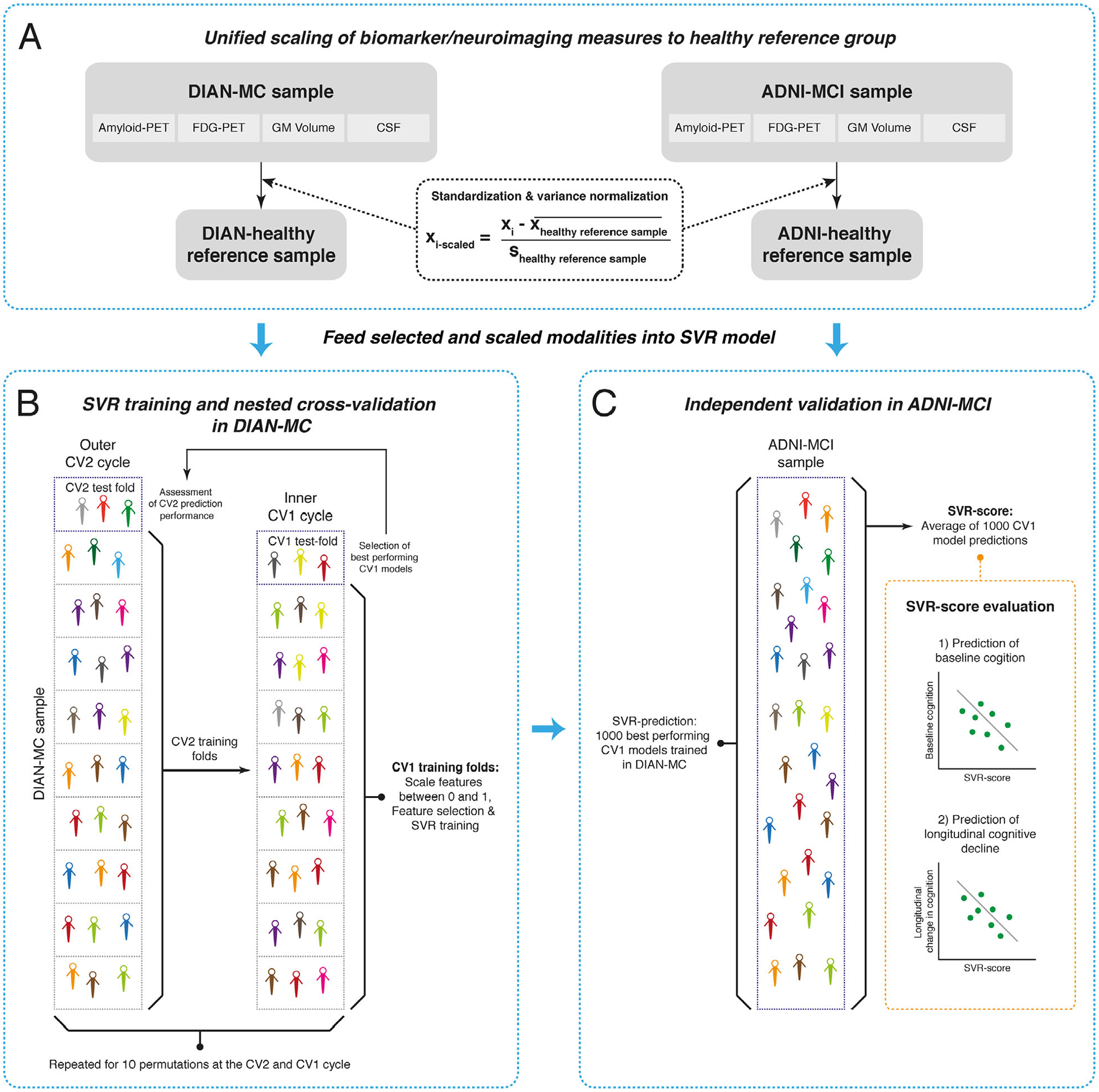
Flow-chart of the support vector regression (SVR) analysis pipeline. (A) Selected data from the DIAN-MC and ADNI-MCI sample are standardized and variance normalized to the respective healthy reference groups to ensure comparability of biomarker scaling across samples. (B) The SVR model is trained based on selected modalities in DIAN-MC in a nested cross-validation framework. (C) The trained SVR-models are blindly applied to the scaled ADNI-MCI biomarker data yielding a SVR score per subject. The SVR score is then evaluated as a predictor of baseline cognition and longitudinal cognitive decline in ADNI

Machine-learning analysis was conducted using NeuroMiner, a Matlab-based machine-learning toolbox (www.pronia.eu/neurominer).^34,35^ In DIAN, we applied SVR to the neuroimaging/biomarker values to predict EYO as a proxy of future cognitive decline. Prior to SVR analysis, all biomarker/neuroimaging values were scaled to a healthy reference sample within the ADNI and DIAN cohort, to yield comparably interpretable values across samples (see Figure 1A and supporting information). SVR training in DIAN (see Figure 1B) was conducted via repeated nested CV to obtain an unbiased SVR performance estimate in data unseen during SVR training. To this end, the DIAN MC group was randomly divided into 10 subsamples (ie, outer CV2-folds). During 10 iterations, a respective outer CV2 test fold was held out, while the remaining nine outer CV2-folds were pooled as SVR training data, and randomly divided into 10 subsamples (inner CV1-folds). For 10 iterations, a respective inner CV1 test fold was held out, while the remaining inner CV1 data were used for SVR parameter tuning. The trained SVR parameters were then applied to the held-out inner CV1 test fold to estimate prediction performance in yet unseen data. For each CV1 iteration, the optimal SVR model was selected based on CV1 prediction performance (ie, minimum mean squared error between actual and SVR-predicted EYO). Last, the selected inner CV1 models were applied to the held-out outer CV2 test fold to estimate the mean CV2 prediction performance. This nested CV approach was complemented by repeated double CV, including 10-fold random permutation of the DIAN MC sample and repeating the above-described procedure for each permutation for CV1 and CV2 levels.^36^ Together, feature selection and parameter optimization were performed on the CV1 level, while final SVR prediction performance was assessed exclusively on CV2 test folds unseen during SVR training (Figure 1B).

To extract the most predictive features (ie, imaging ROI values and CSF data), we performed feature selection at the CV1 level retaining the top 35% of features showing the highest Spearman correlation (ie, to minimize the influence of extreme values or skewed data) with EYO. Overall feature selection probability was defined as the likelihood of a given feature to be included across all optimal CV1 models. Altering the feature selection threshold between 25% and 45% did not change the overall result pattern reported below.

### 2.4 Out-of-sample validation in ADNI

For our main analysis, we applied the 10000 ADAD-trained optimal CV1 SVR models to the ADNI MCI biomarker data, and computed the mean SVR score across all CV1 models, which we hypothesized to predict future cognitive decline (see Figure 1C).

### 2.5 Statistics

Baseline measures were compared between groups using chi-squared tests for categorical variables and analysis of variance (ANOVAs; ADNI) or two-sample *t* tests (DIAN) for continuous measures.

In DIAN MC, we estimated SVR prediction performance for all possible modality combinations, by assessing Pearson-moment correlations between actual and SVR-predicted EYO. Feature selection probabilities were visualized for the SVR model including all modalities (ie, CSF [C], Amyloid-PET [A], FDG-PET [F], and GM [G]).

Next, we assessed whether SVR scores predicted baseline cognition or cognitive decline in the ADNI MCI validation sample. As measures of interest, we used ADNI-MEM, an established memory composite and ADAS13, a measure of global cognition.^37^ Subject-specific cognitive change slopes were determined by fitting subject-specific linear models with ADNI-MEM or ADAS13 scores as the dependent variable and time from baseline as the independent variable. Annual cognitive changes were estimated for baseline to year 1, year 2, year 3, or year 4 for those subjects who had complete annual clinical follow-up data, respectively. Using linear models controlled for age, sex, and education, we tested whether higher SVR scores predicted (1) stronger base line cognitive impairment and (2) faster cognitive decline across 1 to 4-year follow-up. In an additional step, we included baseline cognition as a covariate to assess whether SVR scores predicted cognitive decline after accounting for baseline symptom severity. Analyses in ADNI MCI were stratified by A*β*-status to test whether SVR–based prediction of cognition is specific for MCI-A*β*+ subjects.

Last, we tested whether SVR-based selection of MCI-A*β*+ subjects at risk of cognitive decline enhances the sensitivity to detect intervention effects. Using linear mixed models, we assessed the main effect of time from baseline on cognition (ie, ADNI-MEM or ADAS13) for the entire MCI-A*β*+ sample, or for SVR-selected subsamples of MCI-A*β*+ subjects at highest risk of cognitive decline (ie, showing above median SVR scores), controlling for age, sex, education, and random intercept. Following previous work,^38^ sample size estimates were calculated on group-mean cognitive changes (ie, ADNI-MEM or ADAS13), with hypothetical 10%/20%/30%/40% intervention effects using the R-package *pwr* (settings: two-sample *t* test, two-tailed, type I error rate = 0.05, power = 0.8). Analyses were conducted for the best-performing SVR models with one, two, three, or four modality combinations for 1 to 4-year follow up.

All statistical analyses were performed in R statistical software. Effects were considered significant at a two-tailed alpha threshold of0.05.

## 3 RESULTS

Baseline characteristics of the study samples are presented in Table 1.

### 3.1 SVR training and cross-validation in ADAD

**FIGURE 2:**
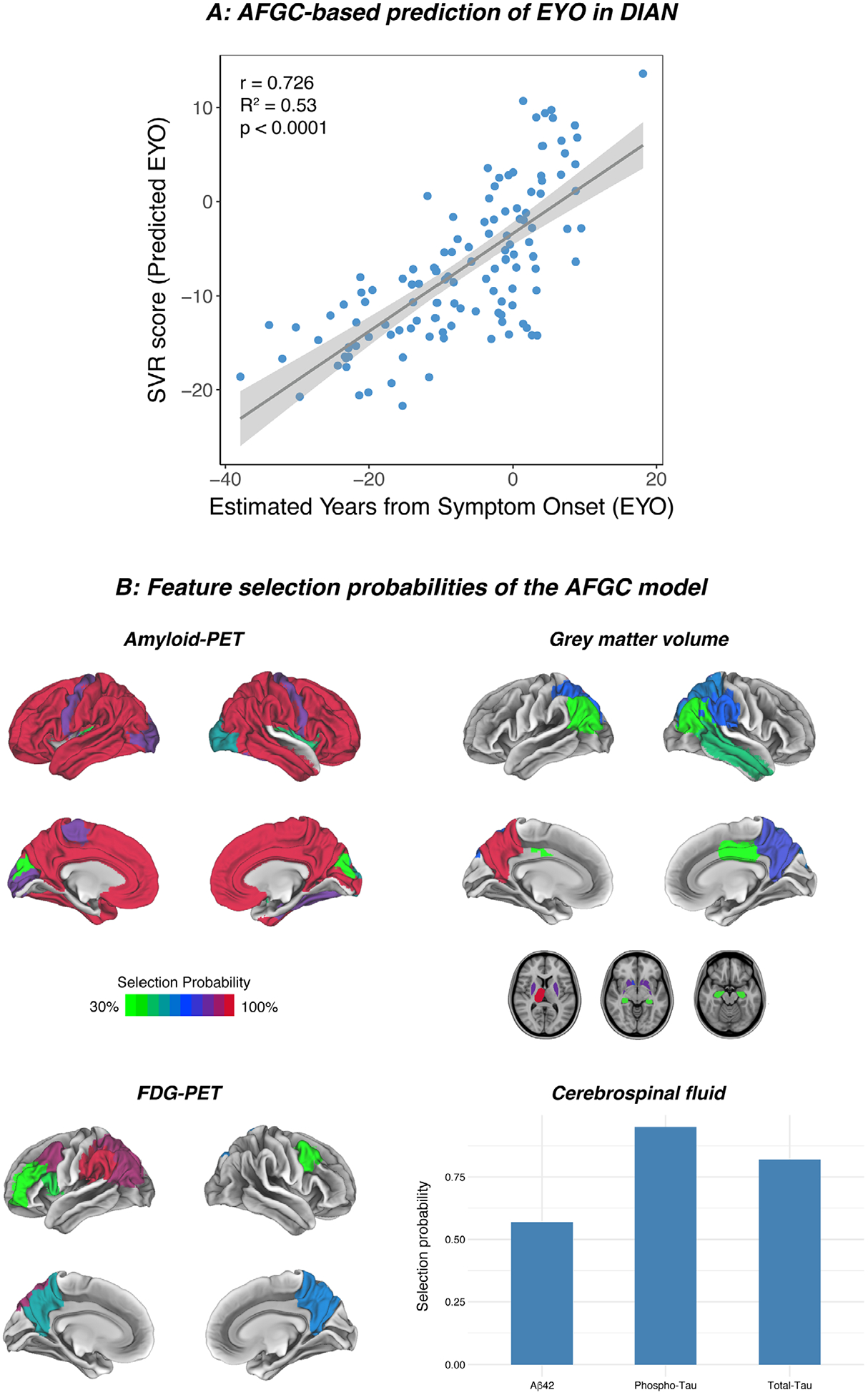
SVR-based prediction of EYO in autosomal-dominant Alzheimer’s disease and feature selection probabilities. (A) Scatterplot showing the association between observed EYO scores and AFGC-predicted estimated years to symptom onset (EYO) scores in DIAN-MC. (B) Selection probabilities of the AFGC model indicate the percentage of final CV1 models (see step in Figure 1B) that included the respective region of interest/biomarker. Features were selected in the DIAN-MC cohort during each CV1 cycle based on the correlation with the outcome measure (ie, EYO)

The SVR model trained on all available modalities (ie, amyloid [A], FDG-PET [F], gray matter [G], CSF [C], abbreviated as AFGC) in DIAN MC accurately predicted EYO at the CV2 level (r = 0.726, *P* <0.0001, R^2^ = 0.53, Figure 2A). This model also showed the numerically highest predictive performance of EYO, compared to all other possible modality combinations (Table S1 in supporting information). For the AFGC model, we mapped the feature selection likelihood for each modality (Figure 2B). For amyloid-PET, almost the entire neocortex showed a high feature selection probability (ie, > 90%). For FDG-PET predominantly medial and lateral parietal and lateral frontal, ROIs showed high feature selection probability. For GM, mainly medial and lateral parietal and medial temporal, ROIs such as the hippocampus showed high feature selection probabilities. For CSF, p-tau_181_ showed the highest selection probability, followed by total tau and A*β*_1−42_. When restricting biomarker modalities for SVR training, the best-performing models for EYO prediction were AFG for three modalities (r = 0.703, *P* <0.0001, R^2^ = 0.49), AG for two modalities (r = 0.677, *P* <0.0001, R^2^ = 0.46), and G for single modality (r = 0.628, *P* <0.0001, R^2^ = 0.39).

### 3.2 Independent validation in ADNI—Predicting cognitive decline in MCI-A*β*+

Next, we applied the ADAD-trained SVR model on all modalities (ie, AFGC) to ADNI MCI biomarker data. As hypothesized, greater AFGC scores predicted lower baseline ADNI-MEM (*t*(211) = −6.692, *β* =−0.410, *P* <0.0001) and ADAS13 scores (*t*(211) = 5.615, *β* = 0.355, *P* <0.0001) in the MCI-A*β*+ group but not in MCI-A*β*− (ADNI-MEM: *t*(170) = −0.239, *β* = −0.017, *P* =0.812; ADAS13: *t*(170) = 0.457, *β* = 0.035, *P* = 0.649).

**FIGURE 3:**
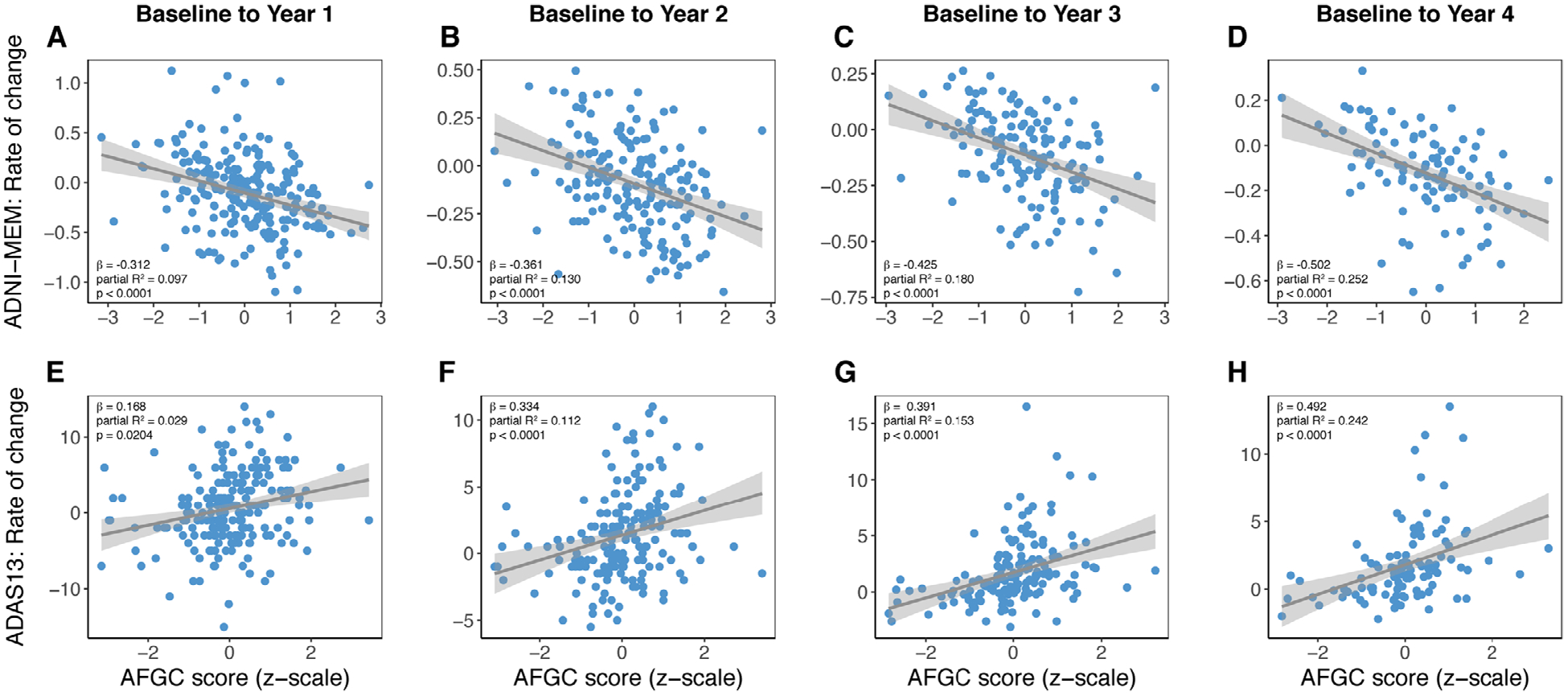
SVR-based prediction of cognitive changes in ADNI MCI-A*β*+. Scatterplots showing the association between AFGC-derived SVR scores and longitudinal cognitive change in ADNI-MCI-A*β*+ for ADNI-MEM (A-D) and ADAS13 (E–H). Standardized *β*-values, partial R^2^, and *P*-values are based on linear regression models adjusted for age, sex, and education

Most importantly, we found higher AFGC scores in MCI-A*β*+ to predict faster decline in ADNI-MEM consistently across all follow up intervals, from baseline to year 1 (*t*(211) = −4.547, *β* = −0.312, *P* <0.0001, Figure 3A), year 2 (*t*(179) = −4.954, *β* = −0.361, *P* <0.0001, Figure 3B), year 3 (*t*(140) = −5.240, *β* = −0.425, *P* <0.0001, Figure 3C), and year 4 (*t*(100) = −5.317, *β* = −0.502, *P* <0.0001, Figure 3D) where partial R^2^-values increased for longer follow-up durations. Similarly, higher AFGC scores predicted decline in ADAS13 from baseline to year 1 (*t*(211) = 2.337, *β* = 0.355, *P* = 0.0204, Figure 3E), year 2 (*t*(179) = 4.510, *β* = 0.334, *P* <0.0001, Figure 3F), year 3 (*t*(140) = 4.756, *β* =0.391, *P* <0.0001, Figure 3G), and year 4 (*t*(100) = 5.143, *β* = 0.492, *P* <0.0001, Figure 3H). No association was found between SVR scores and cognitive changes in MCI-A*β*-suggesting specificity for A*β*+ subjects. All results are summarized in Table 2. Further, all above-listed results remained consistent when additionally controlling for baseline cognition (ie, ADNI-MEM or ADAS13), suggesting that AFGC scores predict cognitive decline independent of baseline symptom severity (see Table S2 in supporting information).

### 3.3 SVR-based selection of MCI-A*β*+ at risk of decline enhances the sensitivity to detect intervention effects

Next, we tested whether SVR-based selection of MCI-A*β*+ subjects at highest risk of cognitive decline (ie, defined as falling above the SVR score median) enhances the sensitivity to detect potential intervention effects. We first determined the main effect of time on longitudinal cognition (ADNI-MEM or ADAS13) in the unselected MCI-A*β*+ versus SVR-selected subsamples using linear mixed models, controlling for age, sex, education, and random intercept. Using the annual cognitive change scores, we estimated the required sample sizes to detect intervention effects of 10% to 40% at 1 to 4-year follow-up with an alpha of 0.05 at a power of 0.8. For the unselected MCI-A*β*+ sample, we found small but significant ADNI-MEM decreases across 1-year follow-up (*β* = −0.052, *P* <0.0001, R^2^ = 0.01). The sample size per arm to detect 10%/20%/30%/40% reduction in cognitive decline was 18984/4301/1737/892. In “at-risk” subjects with above median AFGC scores, we found a stronger effect of time on ADNI-MEM (*β* = −0.109, *P* <0.0001, R^2^ = 0.053). Here, the required N to detect 10%/20%/30%/40% reduction in cognitive decline was greatly reduced to 3375/765/310/159. Congruent results were found when performing these analyses for longer follow-up durations or for the best-performing three-modality, two-modality, and one-modality SVR models (AGC, AG, or G) for selection of at-risk subjects (see Table 3). Congruent results were also found when conducting all above-listed analyses using ADAS13 as measure of cognition (Table 3). Together, SVR-based “at-risk” selection may greatly reduce the required sample size to detect intervention effects.

## 4 DISCUSSION

Our major aim was to establish a biomarker-based machine-learning model for point prediction of AD-related cognitive decline. Our first main finding was that machine-learning (ie, SVR) models trained for predicting future symptom manifestation in ADAD (ie, EYO) generalize well to sporadic AD, where we found high prediction accuracy for 1-to 4-year global cognitive and memory decline. Second, we show that SVR-based selection of sporadic AD subjects at highest risk for cognitive decline can drastically reduce patient numbers required for detecting intervention effects, even when using unimodal biomarkers. Together, our findings suggest that ADAD-informed and biomarker-based machine learning can accurately predict cognitive decline in sporadic AD and can be helpful for subject stratification in clinical trials.

Our findings make a significant contribution for identifying subjects at risk of imminent cognitive decline. Our results are validated across two independent and deeply phenotyped cohorts. A strength of the study is that we chose ADAD for model training, as those subjects are relatively young and age-related comorbidities (eg, small-vessel disease) are minimal.^39^ This allowed extracting AD-specific brain changes that occur early in the disease course. We acknowledge differences between ADAD and sporadic AD including earlier symptom onset, higher likelihood of non-memory symptoms,^40^ and higher prevalence of psychiatric comorbidities^41^ in ADAD versus sporadic AD. However, both sporadic AD and ADAD share core neuropathological features and biomarker abnormalities including amyloid and tau accumulation, FDG-PET hypometabolism, and neurodegeneration.^28,41^ Importantly, the emergence of cognitive symptoms in ADAD is driven by AD pathology rather than age-related comorbidities, and thus, provides a good model for AD-related cognitive decline.^27,42^

The most predictive features in amyloid-PET included almost the entire neocortex, sparing primary sensorimotor, and medial temporal regions that develop amyloid very late in AD.^43,44^ Further, predictive regions included posterior parietal and medial temporal regions for MRI and posterior parietal and lateral frontal regions for FDG-PET, that is, AD-typical predilection sites. Furthermore, the prediction of cognitive decline was non-significant in MCI-A*β*- and was independent of confounding factors including age, sex, education, or baseline cognition. Together, these findings support the validity of our prediction model to depend on key AD-related brain changes.

Previous studies evaluating biomarker combinations^9,12^ or machine learning demonstrated that biomarker combinations can enhance the accuracy of predicting cognitive decline or conversion to dementia.^20,21^ Consistently, we found increasing prediction accuracy as model estimation was informed by more biomarker modalities. We guarded against overfitting by first training and cross-validating the machine-learning algorithm on ADAD,^41^ with subsequent independent validation in sporadic AD. Importantly, in sporadic AD, we found that biomarker-based risk enrichment led to drastically lower sample sizes required to detect intervention effects with cognitive decline as an endpoint. This finding has several implications: First, screening examinations that are frequently used in intervention trials (eg, amyloid-PET and MRI) can be used to enrich for “at-risk” subjects to enhance power and reduce subject numbers and costs for interventions^45^ or to assess whether intervention effects depend on individual progression risk.^46^ Together, knowledge of individual risk for cognitive decline may be used for more adaptive and cost-efficient intervention research, which is urgently needed in view of numerous failed clinical trials in AD.^47^

However, several caveats should be considered when interpreting our results: First, DIAN and ADNI differ in inclusion criteria (ADAD vs sporadic AD) and data acquisition protocols (ie, MRI hardware, PET tracers, and CSF immunoassays), reducing comparability across studies. To address this, we a priori adjusted all biomarker data to healthy control subjects within each study, to obtain harmonized and comparably interpretable values. Importantly, however, rather than pooling data across ADNI and DIAN, where variability across the two studies may hamper model estimation, we used an independent-validation approach for assessing external validity of the ADAD-trained SVR models. The high generalizability from ADAD to sporadic AD supports the robustness and external validity of the SVR models, suggesting that our findings are not driven by assessment procedures or study selection criteria.

Second, the sporadic AD group was restricted to MCI; hence, it remains open whether our findings generalize toward earlier AD stages. However, previous studies have shown that preclinical AD subjects show only little cognitive change across 1 to 4-year follow-up intervals;^48^ hence, we reason that longer follow-ups and large numbers currently unavailable in ADNI are required to test whether the SVR model can predict cognitive changes in preclinical AD.

Third, we trained the model on EYO in ADAD, which is only a cross-sectional proxy of future cognitive decline.^27^ Computing actual cognitive decline in ADAD would have drastically reduced the sample size to those with available longitudinal cognitive data. To maximize sample size, which is critical for training and CV of machine-learning models within the ADAD group, we thus preferred to use cross sectional data.^49^ When sufficient longitudinal data become available in DIAN, future studies may assess whether SVR performance can be further improved when the SVR model is trained on actual longitudinal cognitive decline.

Fourth, due to limited data availability, we did not include tau-PET, which has been recently shown to be a good predictor of future cognitive decline in AD.^15^ However, we included measures of tau-related pathology (ie, CSF), which have been previously shown to correlate with the level of tau-PET uptake^50^ and to predict future cognitive decline.^9^ Nevertheless, inclusion of tau-PET in the SVR model may improve prediction of cognitive decline. Here, our methodological framework will easily allow us to include tau-PET as an additional modality as soon as large enough data become available.

Together, we show that ADAD-informed machine learning can be powerful for point prediction of future cognitive decline in sporadic AD. These findings have important implications because our proposed SVR models may help derive single prognostic indices from increasingly complex biomarker data. The proposed models allow flexible inclusion of various biomarkers, rendering them highly adaptable to individual cohorts. To enhance the external validity of our proposed SVR models, it will be of special importance in the future to determine their prediction performance also across less selective and community-based samples, as well as across different biomarker combinations and acquisition protocols. Our findings may be critical for clinical AD research to identify subjects at risk for progression and to evaluate therapeutic interventions for future cognitive decline.

## Supplementary Material

Supporting Material 1

Supporting Material 2

Supporting Material 3

## Figures and Tables

**TABLE 1:** Baseline demographics

DIAN	MC (N = 121)	NC (healthy reference (N = 54)		*P*
Age	38.66 (9.98)	38.87 (10.48)		0.8998
Sex (m/f)	49/72	19/35		0.5056
Education	14.27 (3.02)	15.50 (2.23)		0.0081
MMSE	27.74(7.88)	30.00 (0.00)		0.0365
Logical-memory	11.44(5.9)	15.78 (3.45)		<0.0001
EYO	−7.10 (11.23)	−9.35 (10.94)		0.2185
ADNI	MCI-A*β*+ (N = 216)	MCI-A*β*− (N = 175)	CN (healthy reference) (N = 49)	*P*
Age	72.74 (6.69)	70.16(7.76)	65.76 (2.69)	<0.0001
Sex (m/f)	117/99	94/81	23/26	0.6345
Education	16.00 (2.79)	16.47 (2.47)	17.14 (2.27)	0.0139
MMSE	27.68(1.83)	28.54 (1.44)	29.51(0.51)	<0.0001
ADAS13	17.18(6.96)	12.21 (5.48)	6.24 (3.83)	<0.0001
ADNI-MEM	0.11 (0.63)	0.62 (0.63)	1.46 (0.61)	<0.0001

Values are displayed as mean (standard deviation). *P*-values for group comparisons are derived from two-sample *t* tests for DIAN and ANOVAs for ADNI for continuous measures and Chi-squared tests for categorical measures. Abbreviations: EYO, estimated years to symptom onset; MC, mutation carrier; MMSE, Mini-Mental State Exam; NC, non-carrier

**TABLE 2:** Prediction of baseline cognition and longitudinal cognitive changes in ADNI MCI-A*β*+

	ADNI MCI-A*β*+				
	N	*β*	T	*P*	Partial R^2^
ADNI-MEM					
Baseline	216	−0.410	−6.692	<0.0001	0.168
Year 1	216	−0.312	−4.547	<0.0001	0.097
Year 2	184	−0.361	−4.954	<0.0001	0.130
Year 3	145	−0.425	−5.240	<0.0001	0.180
Year 4	105	−0.502	−5.317	<0.0001	0.252
ADAS-13					
Baseline	216	0.355	5.615	<0.0001	0.126
Year 1	216	0.168	2.337	0.0204	0.028
Year 2	184	0.334	4.510	<0.0001	0.112
Year 3	145	0.391	4.746	<0.0001	0.153
Year 4	105	0.492	5.143	<0.0001	0.242

All regression models were controlled for age, sex, and education.

**TABLE 3:** Sample size estimation for detecting intervention effects based on unselected MCI-A*β*+ subject or on SVR selection of at-risk subjects (defined as falling above the median of SVR scores)

		Main effect of time on cognitive changes in MCI-A*β*+				Required N per arm to detect an intervention effect of				
Cognitive test	Group selection	*β*	T	*P*	Partial R^2^	10%	20%	30%	40%	% Reduction of required N
1-year follow-up										
ADNI-MEM	No selection	−0.052	−4.021	<0.0001	0.010	18984	4301	1737	892	NA
	G-risk	−0.084	−4.703	<0.0001	0.028	6937	1572	635	327	63%
	AG-risk	−0.090	−5.170	<0.0001	0.033	5742	1301	526	271	70%
	AGC-risk	−0.095	−5.574	<0.0001	0.037	4940	1120	458	233	74%
	AFGC-risk	−0.109	−6.744	<0.0001	0.053	3375	765	310	159	82%
ADAS13	No selection	0.037	1.764	0.0792	0.002	94800	21475	8672	4453	NA
	G-risk	0.070	2.317	0.0224	0.008	27638	6261	2529	1299	71%
	AG-risk	0.073	2.326	0.0219	0.009	27392	6205	2506	1287	71%
	AGC-risk	0.089	2.933	0.0041	0.013	17335	3927	1586	815	82%
	AFGC-risk	0.100	3.149	0.0021	0.016	15086	3418	1381	709	84%
2-year follow-up										
ADNI-MEM	No selection	−0.076	−6.425	<0.0001	0.026	9166	2077	839	431	NA
	G-risk	−0.131	−8.071	<0.0001	0.080	2741	622	252	130	70%
	AG-risk	−0.136	−8.372	<0.0001	0.085	2574	584	236	122	72%
	AGC-risk	−0.140	−8.862	<0.0001	0.095	2370	538	218	112	74%
	AFGC-risk	−0.144	−8.850	<0.0001	0.097	2293	520	211	109	75%
ADAS13	No selection	0.122	6.421	<0.0001	0.031	8748	1982	801	412	NA
	G-risk	0.178	6.208	<0.0001	0.060	4360	988	400	206	50%
	AG-risk	0.178	6.137	<0.0001	0.060	4545	1030	417	214	48%
	AGC-risk	0.199	6.957	<0.0001	0.075	3526	799	323	166	60%
	AFGC-risk	0.209	7.160	<0.0001	0.081	3410	773	313	161	61%
4-year follow-up										
ADNI-MEM	No selection	−0.173	−10.755	<0.0001	0.107	3857	874	354	182	NA
	G-risk	−0.264	−12.331	<0.0001	0.250	1348	306	124	64	65%
	AG-risk	−0.291	−14.188	<0.0001	0.307	905	206	84	43	76%
	AGC-risk	−0.294	−14.303	<0.0001	0.318	900	205	83	43	76%
	AFGC-risk	−0.264	−12.694	<0.0001	0.256	1295	294	119	62	66%
ADAS13	No selection	0.239	10.750	<0.0001	0.118	3818	866	350	180	NA
	G-risk	0.325	10.943	<0.0001	0.222	1778	404	163	84	53%
	AG-risk	0.356	11.921	<0.0001	0.348	1462	332	135	69	62%
	AGC-risk	0.369	11.919	<0.0001	0.262	1441	327	133	68	62%
	AFGC-risk	0.338	11.302	<0.0001	0.235	1728	392	159	82	54%

## RESEARCH IN CONTEXT

1. Systematic review: Prodromal Alzheimer’s disease (AD) patients are at increased risk to develop AD dementia. However, there are considerable differences in cognitive decline rates between individuals, posing challenges for clinical prognostication. While AD biomarkers are established for diagnostics, there is an unmet need of biomarker models for predicting the rate of future cognitive decline.
2. Interpretation: Machine learning applied to complex AD biomarker data (ie, neuroimaging and biofluid) accurately predicted estimated symptom onset in autosomal dominant Alzheimer’s disease and generalized well to an independent sample of sporadic AD patients for predicting 1 to 4-year cognitive decline. Machine learning–based selection of at-risk patients with sporadic prodromal AD significantly reduced numbers required for detecting intervention effects by up to 50% to 75%. These findings suggest that machine learning may help derive meaningful prognostic indices from increasingly complex biomarker data.
3. Future direction: Does the addition of tau-positron emission tomography and neuroinflammatory biomarkers further increase prediction accuracy of future cognitive decline?
