# Supplementary material for: Predicting sporadic Alzheimer’s disease progression via inherited Alzheimer’s disease-informed machine-learning
Source: Alzheimers Dement. Author manuscript; Available in PMC 2021 Mar 1. (PMC7222030; doi:10.1002/alz.12032)
Supplement: Supporting Material 1 [file NIHMS1565951-supplement-Supporting_Material_1.docx]

***SUPPLEMENTARY***

***Supplementary methods***

***Harmonizing neuroimaging and biomarker data across DIAN and ADNI***

While the DIAN and ADNI study protocols share most technical aspects of neuroimaging and biomarker acquisition, differences remain in the selection of amyloid-PET tracers (PiB-PET in DIAN vs. AV45 in ADNI) and CSF assays (xMap in DIAN vs. Elecsys in ADNI), which may impede comparability of absolute biomarker/imaging values across studies. Further, the AD target populations of DIAN (i.e. MC) and ADNI (i.e. MCI) differ in age-range (20-61 years in DIAN vs. 55-91 years in ADNI), hence other age-related brain changes (e.g. non-AD related grey matter atrophy, TDP-43, small-vessel disease etc.) that may be unrelated to core AD pathophysiology are likely pronounced in the ADNI cohort and potentially compromise between-sample comparability. To increase the comparability across ADNI and DIAN samples, we scaled all neuroimaging/biomarker values to a respective healthy reference group within each study, yielding comparably interpretable scores for both ADNI and DIAN. The idea for harmonizing all biomarkers across samples is based on the recent development of the Centiloid scale, which was developed to compare amyloid biomarkers (i.e. PET) uptake across different acquisition protocols (i.e. tracers & scanners) [1]. Specifically, we applied standardization and variance normalization of each neuroimaging ROI value and CSF biomarker within the AD target groups (i.e. DIAN MC and ADNI MCI) to the respective healthy reference groups. In both DIAN and ADNI, this reference group was selected from the entire study sample based on normal cognition (diagnosed as cognitively normal & MMSE>28) and normal amyloid levels (DIAN: non-carrier & global PiB SUVR<1.19; ADNI: global AV45 SUVR<1.11). In ADNI, the reference group was further restricted to an age below 70 years, to minimize confounding effects of possible non-Alzheimer’s age-related brain pathologies. Characteristics of the AD target and healthy reference groups are shown in table 1. Within the DIAN MC and the ADNI MCI groups each neuroimaging/biomarker score was then scaled to the 95% trimmed mean expressed in units of standard deviation of the respective healthy reference group using the following linear z-score transformation:

$$X_{i}scaled= \frac{x_{i}- \bar{x_{Reference (95\% trimmed)}}}{\sigma_{x-Reference}}$$

Where *x_i_* is the biomarker/neuroimaging value of a given subject *i*, $\bar{x_{Reference (95\% trimmed)}}$is the 95% trimmed mean of the biomarker/neuroimaging value in the healthy reference group and $\sigma_{x-Reference}$ is the standard deviation of the biomarker/neuroimaging value distribution of *x* in the healthy reference group. Accordingly, in both DIAN MC and ADNI MCI, values > 0 indicate a biomarker increase compared to the respective reference group (e.g. abnormal Amyloid-PET, CSF-tau), whereas values < 0 indicate a decrease (e.g. abnormal CSF-Aβ, grey matter volume, FDG-PET metabolism). Note that we used the 95% trimmed mean, to ensure that scaling was robust against potential outliers/extreme values within the healthy reference groups.

Prior to applying machine learning all scaled features were further transformed to a range between 0 and 1, following common machine learning standards. The above described unified scaling procedure is schematically illustrated in Figure 1A. Note that exploratorily running the entire machine-learning pipeline (see description below) with raw biomarker scores across DIAN and ADNI yielded overall congruent results to those reported within the manuscript.

***Neuroimaging & biomarker assessment:***

*DIAN:*

For each imaging modality, we obtained FreeSurfer (Version 5.1) based cortical ROI values in Desikan Killiany Atlas space as provided by the DIAN imaging core, i.e. 3 T T1-MRI-based cortical thickness measures as well as PiB-PET and FDG-PET SUVR scores. For grey matter, also FreeSurfer derived volumes of subcortical structures (i.e. hippocampus and basal ganglia) were also included. All PET SUVR values were intensity normalized to established reference regions (whole cerebellum for PiB-PET & brainstem for FDG-PET). CSF concentrations of Aβ_1-42_, phosphorylated tau at threonine 181 (p-tau_181_) and total tau were measured by the DIAN biomarker core using multiplex xMap Luminex technology. For details on processing of these data please see [2, 3].

*ADNI:*

Neuroimaging in ADNI has been conducted using similar protocols as in DIAN which was described in detail previously [4, 5]. Analogous to DIAN, we obtained FreeSurfer (Version 5.1) based ROI values in Desikan Kiliany space as provided by the ADNI imaging core for T1-MRI-based cortical thickness and AV45-PET. Since FDG-PET values were not provided in Desikan-Killiany space from the ADNI imaging core, we applied the same previously described fully-automated FreeSurfer-based pipeline from DIAN [2] to the ADNI FDG-PET images in order to obtain matching ROI values. In brief, we applied fully automated FreeSurfer parcellation (i.e. Desikan Killiany Space) to 3T T1-MRI scans. The resulting ROI parcellation was applied to co-registered FDG-PET images to extract mean FDG-PET values. Congruent with the PET intensity normalization procedure in DIAN, ADNI PET images were intensity normalized to the same reference regions (whole cerebellum for AV45-PET & brainstem for FDG-PET). CSF biomarkers (Aβ_1-42_, p-tau, total Tau), were analyzed by the ADNI biomarker core using the novel fully automated Electrochemiluminescence (Elecsys) cobas e 601 instrument [6].

**Supplementary References**

[1] Klunk WE, Koeppe RA, Price JC, Benzinger TL, Devous MD, Sr., Jagust WJ, et al. The Centiloid Project: standardizing quantitative amyloid plaque estimation by PET. Alzheimers Dement. 2015;11:1-15 e1-4.

[2] Benzinger TL, Blazey T, Jack CR, Jr., Koeppe RA, Su Y, Xiong C, et al. Regional variability of imaging biomarkers in autosomal dominant Alzheimer's disease. Proceedings of the National Academy of Sciences of the United States of America. 2013;110:E4502-9.

[3] Bateman RJ, Xiong C, Benzinger TL, Fagan AM, Goate A, Fox NC, et al. Clinical and biomarker changes in dominantly inherited Alzheimer's disease. N Engl J Med. 2012;367:795-804.

[4] Jack CR, Jr., Barnes J, Bernstein MA, Borowski BJ, Brewer J, Clegg S, et al. Magnetic resonance imaging in Alzheimer's Disease Neuroimaging Initiative 2. Alzheimers Dement. 2015;11:740-56.

[5] Jagust WJ, Landau SM, Koeppe RA, Reiman EM, Chen K, Mathis CA, et al. The Alzheimer's Disease Neuroimaging Initiative 2 PET Core: 2015. Alzheimers Dement. 2015;11:757-71.

[6] Bittner T, Zetterberg H, Teunissen CE, Ostlund RE, Jr., Militello M, Andreasson U, et al. Technical performance of a novel, fully automated electrochemiluminescence immunoassay for the quantitation of beta-amyloid (1-42) in human cerebrospinal fluid. Alzheimers Dement. 2016;12:517-26.

*Supplementary table 1: Comparison of SVR performance in DIAN when using different modality combinations*

|  | | *Correlation between EYO and SVR scores* | | | |
| --- | --- | --- | --- | --- | --- |
| *Modality combinations* | | *r* | *95% CI* | *p* | *R^2^* |
| *AFGC* | | *0.726* | *0.64;0.80* | *<0.0001* | *0.53* |
| *AFG* | | *0.703* | *0.60;0.78* | *<0.0001* | *0.49* |
| *AFC* | | *0.626* | *0.50;0.72* | *<0.0001* | *0.39* |
| *AGC* | | *0.716* | *0.62;0.79* | *<0.0001* | *0.51* |
| *FGC* | | *0.696* | *0.59;0.78* | *<0.0001* | *0.48* |
| AF | | *0.588* | *0.46;0.69* | *<0.0001* | *0.35* |
| *AC* | | *0.534* | *0.39;0.65* | *<0.0001* | *0.29* |
| *AG* | | *0.677* | *0.57;0.76* | *<0.0001* | *0.46* |
| *FG* | | *0.647* | *0.53;0.74* | *<0.0001* | *0.42* |
| *FC* | | *0.607* | *0.48;0.71* | *<0.0001* | *0.37* |
| *GC* | | *0.706* | *0.60;0.79* | *<0.0001* | *0.50* |
| *A* | | *0.521* | *0.38;0.64* | *<0.0001* | *0.27* |
| *F* | | *0.503* | *0.36;0.63* | *<0.0001* | *0.25* |
| *G* | | *0.628* | *0.51;0.72* | *<0.0001* | *0.39* |
| *C* | | *0.560* | *0.42;0.67* | *<0.0001* | *0.31* |
|  | *A = Amyloid-PET, F = FDG-PET, G = Grey Matter Volume, C = CSF, 95%CI = 95% Confidence Interval* | | | | |

*Supplementary table 2: Prediction of longitudinal cognitive changes in ANDI MCI Aβ+, controlling for age, gender, education and baseline cognition*

|  | ***ADNI MCI Aβ +*** | | | | |
| --- | --- | --- | --- | --- | --- |
|  | *N* | *β* | *T* | *P* | *Partial R^2^* |
| ***ADNI-MEM*** |  |  |  |  |  |
| *Year 1* | *216* | *-0.247* | *-3.360* | *0.0009* | *0.061* |
| *Year 2* | *184* | *-0.257* | *-3.382* | *0.0009* | *0.066* |
| *Year 3* | *145* | *-0.305* | *-3.532* | *0.0005* | *0.093* |
| *Year 4* | *105* | *-0.422* | *-4.043* | *0.0001* | *0.178* |
| ***ADAS-13*** |  |  |  |  |  |
| *Year 1* | *216* | *0.213* | *2.756* | *0.0064* | *0.045* |
| *Year 2* | *184* | *0.274* | *3.461* | *0.0006* | *0.075* |
| *Year 3* | *145* | *0.299* | *3.425* | *0.0008* | *0.089* |
| *Year 4* | *105* | *0.413* | *4.089* | *<0.0001* | *0.171* |
